# Expectations about pain management after discharge from total hip and knee replacement surgery: a qualitative study with patients and prescribers

**DOI:** 10.3389/fpain.2025.1647020

**Published:** 2025-09-24

**Authors:** Ian Liang, Peter Youssef, Abby Haynes, Chung-Wei Christine Lin, Ian Harris, Mark Horsley, Caitlin M. P. Jones

**Affiliations:** ^1^ Department of Rheumatology, Royal Prince Alfred Hospital, Institute of Rheumatology and Orthopaedics, Sydney, NSW, Australia; ^2^ School of Public Health, The University of Sydney, Sydney, NSW, Australia; ^3^ Sydney Local Health Disctrict, Institute for Musculoskeletal Health, Sydney, NSW, Australia; ^4^ Ingham Institute for Applied Medical Research, Liverpool, NSW, Australia; ^5^ Department of Orthopaedics, Royal Prince Alfred Hospital, Institute of Rheumatology and Orthopaedics, Sydney, NSW, Australia

**Keywords:** pain, total knee and hip arthroplasty, opioid, qualitative, post op

## Abstract

**Objective:**

This study aimed to explore the perspectives of both patients and prescribers regarding analgesia after discharge following total hip or knee replacement surgeries, focusing on opioid use and the factors influencing patient and prescriber decision making.

**Methods:**

Semi-structured interviews were conducted with 20 prescribers and 13 patients. 6 patients were interviewed before and after surgery. Thematic analysis of the data was conducted by three researchers.

**Results:**

For prescribers, three key themes were identified: (1) A patchwork of prescribing practices with diverse influences on health professional’s decisions, including the ‘norm’ of each site; (2) What counts as evidence for practice? in which prescribers relied on clinical experience, more than guidelines; And (3) Risk-benefit trade-offs that prescribers make when challenged to treat pain while minimising side effects. Analysis of patients’ data also identified three key themes: (1) (Unfulfilled) expectations of careful pain management such as evidence-based decisions and close monitoring to avoid harm; (2) Risk-benefit trade-offs: fear of pain vs. fear of side effects including anxiety about both expected pain and expected side effects, and (3) Variation in self-management of opioid use due to varying interpretation of vague instructions. The views and expectations of prescribers and patients differ and sometimes conflict with each other.

**Conclusion:**

This research highlights the need for improved guidance for both prescribers and patients, and clearer communication to optimise the management of pain after discharge.

## Introduction

Major musculoskeletal (MSK) surgeries, such as total hip replacement (THR) and total knee replacement (TKR), often result in severe post-operative pain, which can be difficult to manage. This pain needs to be managed, but at the same time, clinicians must balance the benefits against the risks associated with opioid use, including addiction and misuse (1).

The over-prescription of post-surgical opioid analgesia remains one of the primary contributors to the ongoing opioid crisis, despite some reduction in recent years due to opioid stewardship initiatives (2). There is significant variability in how analgesia is prescribed following THR and TKR (3). In Australia, discharge opioid analgesia can be managed by a variety of different specialties including orthopaedics, anaesthetics, geriatrics, rehabilitation physicians, and junior medical officers. Patients are frequently discharged with a combination of analgesics, including non-opioids and opioids, in varying doses and durations. This variability is influenced by a range of factors, including clinical experience, type of surgical procedure, patient demographics, and baseline opioid use (4). Understanding the broad range of factors that influence prescribing is essential for optimising post-surgical pain management strategies.

While opioid analgesia remains a standard treatment for post-surgical pain management, there is evidence that non-opioid medications can be equally effective, especially for less invasive surgeries (5, 6). Non-steroidal anti-inflammatory drugs (NSAIDs) and acetaminophen are commonly recommended as first-line analgesics, with opioids prescribed primarily for breakthrough pain (1). The PROSPECT guidelines (7, 8), the Australian and New Zealand College of Anaesthetists (ANZCA) Position statement (9) and the Australian Commission on Safety and Quality in Health Care (ACSQHC) Acute Pain Management Clinical Care Standard (10) provides recommendations for managing pain after surgery advocate for a multimodal approach and that discharge prescriptions. They state that should be based on opioid use in the 24 h prior to discharge should consist of no more than 7 days’ worth of immediate release opioid analgesia on discharge. This advice is not based on direct evidence as there are no trials assessing the role of discharge prescriptions based on prior 24 h usage. The guidelines are also not specific to analgesia following major surgery.

This gap in guidance leaves prescribers with little direction regarding the optimal regimen of opioid to administer at discharge after surgery. To reduce opioid over-prescription, there is a need to consider the perspectives of both prescribers and patients when evaluating post-surgical pain management practices. Therefore, the aim of this study was to explore the motivators and concerns about pain medicine use, especially opioids, on discharge from total knee and total hip replacement surgeries from both a prescriber and patient perspective.

## Patients and methods

### Study design

This study used a qualitative descriptive methodology aimed at providing a straightforward description of participants views and experiences, including the extent to which these perspectives aligned or differed (11). We received ethical approval from the University of Sydney Human Ethics Board (approval no 2023/HE000468).

### Recruitment and informed consent

Recruitment occurred between the periods of December 2023 and December 2024. The study recruited from two populations: (1) medical doctors who prescribed analgesia to patients at discharge after total hip or knee replacement (‘prescribers’), and (2) patients who were scheduled for a total hip or knee replacement. Recruiting participants who were yet to have their surgery allowed investigation of their concerns and expectations without the benefit of hindsight. Recruitment strategies included advertising on targeted social media, leveraging existing surgical and consumer contacts in the research team, and snowball recruitment if opportunities arose, e.g., across inter-organisational professional or consumer networks, and within support groups.

We aimed to sample purposively within each participant group. For prescribers, we sought variation in gender, age, profession, career stage, geographic location (metro, regional, rural) and work settings (public, private). Once data saturation occurred for interviews with a particular demographic of clinicians (e.g., orthopaedic surgeons), we advertised purposively to another demographic (e.g., anaesthetists) to obtain a representative opinion. For patients, we sought variation in durations of relevant musculoskeletal conditions, prior use of opioids (opioid-naive, have used previously, current user, etc.), geographic locations (metro, rural, regional), and health systems (public or private system). Prospective participants who responded to the contact or advertisements were provided with the participant information statement and invited to complete an online consent form and a screening form to collect demographic information (Supplementary Appendix 1, 2).

### Inclusion criteria

Prescribers were eligible if they:

1. Were registered medical professionals practicing in Australia.
2. Had prescribed analgesia to at least 5 patients discharged following total knee or hip replacements within the previous 12 months.

Patients were eligible if they:

1. Were scheduled or on the waiting list for a total hip or knee replacement surgery.
2. Were at least 18 years of age.

### Sample size

In line with qualitative research principles, no *a priori* sample size was specified. Recruitment continued until interviews were not producing new information or perspectives and the researchers determined that data saturation was reached (12).

### Data collection

The semi-structured interviews were used to elicit rich information from participants. Prompts in the interview guide (Supplementary Appendix 3, 4) were loosely informed by the Theoretical Domains Framework (TDF) to ensure that different potential influences on views and behaviours were considered (13). One-on-one interviews were conducted, recorded and auto transcribed via Zoom software. Transcripts were corrected by the interviewer. Confidentiality and anonymity were maintained by de-identifying transcripts and storing all data securely. A running memo was kept by each researcher who recorded key impressions and potential coding categories following each interview (14). Selected patients, aiming to cover a range of demographics, were invited to be re-interviewed after their surgery to explore whether their expectations matched their experience. Clinician interviews were conducted by a rheumatology advanced trainee (IL). All patient interviews were conducted by a researcher with an expertise in patient interviewing and qualitative analysis (CJ).

### Data analysis

Transcripts were analysed in NVivo software (15). The main topics raised in the interviews were identified and coded. These codes were organised into categories and further summarised to come to 3 overarching themes per participant group (16). Three researchers (IL, CJ & AH) independently coded early transcripts and negotiated two coding frames: one for prescriber and one for patient data. The coding frame was applied to further transcripts and modified in response to variations in subsequent data. The final coding categories were then reviewed by the research team to identify descriptive themes that were present across the data sets. Areas of difference and similarity between patients’ pre- and post-surgery data, and between the prescriber and patient data sets, were highlighted. Analysis began soon after data collection commenced, enabling concurrent monitoring of data saturation. Recruitment ceased when the research team agreed that interviews were not adding significant new information.

## Results

Twenty prescribers and thirteen patients were interviewed. Interview duration ranged between 15 min and 1 h. Six of these patients also took part in post-surgical follow up interviews. Most of the prescribers were recruited from personal contacts and the majority agreed to participate when approached. The patients were recruited from a variety of sources including social media and as a result it is difficult to be prescriptive as to the percentage of patients who agreed to participate as to the number of patients approached. Prescriber and patient characteristics can be found in Tables 1, 2. The study included 20 prescribers from diverse clinical backgrounds including clinicians from anaesthetics, orthopaedics, geriatrics and rehab with of varying levels of seniority. Most of the patients were women aged 60–79 years, had hip or knee osteoarthritis, and reported moderate to severe pain despite varied opioid use histories and healthcare settings.

**Table 1:** Participant characteristics table (prescribers only).

Characteristic	*N* = 20
Sex	Male = 14
	Female = 6
Profession	Anaesthetist = 4
	Anaesthetics registrar/fellow = 3
	Orthopaedic surgeon = 2
	Orthopaedic registrar = 1
	Orthopaedic Junior Medical Officer = 5
	Geriatrician = 3
	Rehabilitation consultant = 2
Location of practice	Sydney Local Health District, NSW = 12
	Blacktown, NSW = 4
	Wollongong, NSW = 2
	St George, NSW = 1
	Brisbane, QLD = 1
Type of practice (private vs public)	Public only = 10
	Private + public = 10
	Private only = 0

NSW, New South Wales; QLD, Queensland.

**Table 2:** Participant characteristics table (patients only).

Characteristic	*N* = 13
Gender	Woman = 9/Man = 4
Age (yrs)	50–59 = 1
	60–69 = 5
	70–79 = 6
	80–89 = 1
State of residence	NSW = 3
	VIC = 1
	QLD = 3
	NT = 1
	Did not answer = 5
Born in Australia	Yes = 8/No = 5
Joint being replaced	Hip = 6/Knee = 5/Both = 2
Median duration of pain in affected joint	1.75 years (range 0.5–20)
Reason for joint replacement^a^	Osteoarthritis = 12/Other = 2/Injury = 1
Previous use of opioids	Yes, currently = 4/Yes, in last 90 days = 2/No = 7
Aboriginal or Torres Strait Islander	Aboriginal = 1/Neither = 12
Average pain over previous week (0–10)	Mean (SD) = 6.7 (2.1)/Median (range) = 7 (2–10)
Healthcare system	Public = 8/Private = 5

SD, standard deviation; NSW, New South Wales; VIC, Victoria; QLD, Queensland; NT, Northern Territory.

^a^ Could select more than one option

### Overview of themes

**Figure 1:**
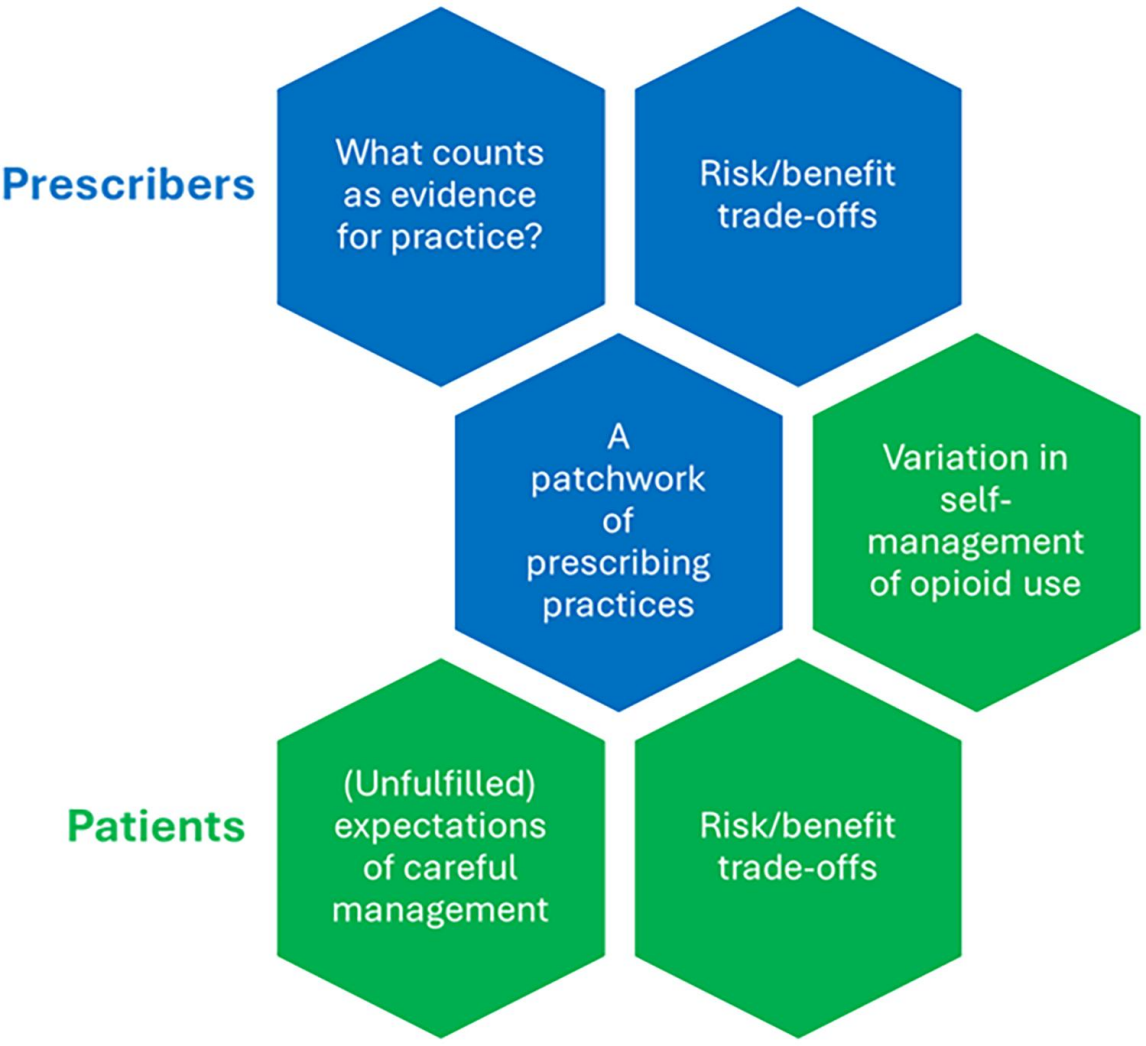
Overview of key themes.

An overview of key themes can be found in Figure 1.

### Prescriber perspective themes

Detailed information about the prescriber themes, subthemes and supportive quotes can be found in Supplementary Appendix 5. They are summarised below.

### Theme 1: a patchwork of prescribing practices

There is a considerable amount of variability in the prescribing practices between clinicians, as noted by a geriatrician who commented on variability of prescribing even between consultants within a specialty “*There's a lot of variety in analgesic prescribing because the patient is under the orthopaedic team, and they have different preferences. Where I'm working, for a particular surgeon for their total knee replacements there's a strict protocol that they want, pregabalin, Panadol and others”*.

#### What types of analgesia work

There was significant variation in post-operative pain management. Some surgeons followed strict protocols, while others deferred to anaesthetists or pain teams. Most patients received simple analgesia with regular paracetamol and a long-acting NSAID like celecoxib or meloxicam. Medications like pregabalin or gabapentin were preferred as an adjunct by some clinicians for neuropathic pain because of perceived fewer side effects. Some clinicians recommended adjunct complementary therapies like fish oil and turmeric, despite admitting that their effectiveness remained unproven. Ice packs were commonly used in hospital and at home. While most patients received additional opioids prescribed on an ‘as required’ basis, prescribing practices varied in terms of the type of opioid, dose, frequency and duration.

#### How much analgesia to dispense on discharge

The number of opioid tablets prescribed on discharge varied. Some junior medical officers prescribed 10–15 tablets of oxycodone 5 mg or tapentadol 50 mg, while others prescribed up to 20 tablets, particularly for early discharges. One clinician explained that they routinely prescribed 20 tablets for practical reasons as there are 20 tablets in a box of oxycodone. The instructed frequency of use varied between 3- and -12-hourly, although the clinician would often advise patients to use less than the maximum if they were able to manage their pain. Opioid use duration was seen to depend on recovery. Anaesthetists typically limited opioids to one week's supply before transitioning to non-opioid relief. Others extended use for up to six weeks, particularly after knee replacements which they considered to be more painful. Clinicians explained that challenges arose when patients had unrealistic expectations of how effectively joint replacement surgery would relieve their pain, resulting in prolonged use of opioids in some cases.

#### Timing of when to take the analgesia

Clinicians saw the role of post-surgical pain relief to support rehabilitation, improve function, and enable sleep, rather than just eliminate pain. They reported that timing of dosing was important, with opioids often given before activities such as physical therapy or at night to promote sleep taking advantage of the side effect of drowsiness.

#### Private vs. public variation

There were prescribing differences between private and public hospitals. Clinicians describe seeing higher opioid dose prescriptions in private hospitals due to perceptions of greater patient expectation of pain relief. In contrast, public hospital clinicians were treating patients with more advanced disease who had been waiting for surgery for longer periods of time. Clinicians argued that these patients were more accustomed to managing pain and could thus manage post operatively with fewer opioids due to a higher pain threshold. In private hospitals, anaesthetists typically handled discharge prescriptions, whereas in public hospitals, junior medical officers (JMOs) were responsible for the majority of prescriptions. In both hospital settings, there was a lack of consistency regarding analgesic prescribing with individual variations as well as variations between disciplines. The higher cost of tapentadol resulted in it being excluded from the formulary in one public hospital, leading to more frequent use of less expensive alternatives like oxycodone. Public patients usually received specialist pain clinic follow-up care, whereas private patients tended to rely on general practitioners (GPs) for analgesia post-operatively. One orthopaedic surgeon noted that GPs often lacked adequate training in post-surgical management and may feel disempowered to reduce opioids, especially when they are not involved from the start of the patient's treatment.

### Theme 2: what counts as evidence for practice?

#### Variable use of guidelines

Clinical guidelines on post-surgical pain management, particularly regarding opioids, were often criticised by senior clinicians for being based on low-quality evidence. Some anaesthetists, recognising this limitation, often made decisions based on personal experience rather than guidelines as noted in “*The issue with hip and knee replacements is that there's a lot of literature out there, but a lot of it is low-quality evidence, so it can be hard to find definitive guidance.”* Despite being responsible for most opioid prescriptions on discharge in public hospitals, JMOs in our study were less familiar with the latest guidelines. However, some core messages from guidelines still influenced prescribing, especially the preference for atypical opioids such as Tapentadol and for using the lowest effective opioid dose.

#### Practice-based knowledge

Many clinicians conveyed that they relied on professional experience or guidance from colleagues when making prescribing decisions. Preferences for or against certain types of analgesia such as NSAIDs or opioids often stemmed from previous encounters with side effects or complications in patients. Some senior clinicians preferred slow-release opioids for long-lasting pain relief despite acknowledging that guidelines advised against their use. JMOs, who often have limited experience managing pain after joint replacements, rely on their registrars or prior experience prescribing analgesics during non-orthopaedic rotations. Senior anaesthetists had found advances with regional techniques and medications like tranexamic acid to have reduced overall opioid use.

### Theme 3: risk/benefit trade-offs: pain, side effects and patient expectations

Clinicians identified the key challenge in post-surgical pain management as balancing pain relief with managing side effects, particularly opioid tolerance and the risk of addiction. Geriatricians were especially cautious with opioids due to concerns about sedation and delirium, which can worsen patient outcomes. They noted however, that untreated pain itself can also cause delirium. The risk-benefit assessment depended on patient-specific factors such as chronic pain, comorbidities and overall health. For example, NSAIDs like meloxicam were used safely in patients with normal renal function, but alternatives like hydromorphone may be preferred in those with renal disease. A patient's age, weight and co-morbidities such as sleep apnoea—which raises the risk of respiratory depression—were also important considerations. Clinicians did not tend to reduce opioids if side effects could be managed with other measures such as aperients for constipation. Surgical factors, such as recovery from total knee replacements being viewed as more painful than total hip replacement, lead to increased dosage and duration of opioid prescriptions.

Clinicians observed that patient expectations significantly influenced their risk-benefit assessments. Some patients, particularly those with high expectations of minimal or no pain, may request more opioids, leading to overuse. Anxious patients or those with prior negative experiences with pain, often demanded more opioids and reported higher pain levels, possibly due to a lower pain threshold, as noted by an anaesthetist “*Those patients who are more anxious, they tend to feel that they require more opioid analgesic agents. They're often the ones that get into trouble with adverse effects”*. One geriatrician noted that orthopaedic surgeons played a significant role in managing expectations as patients are more likely to follow pain management plans when surgeons emphasise the need to reduce opioid use when appropriate.

### Addressing challenges

To address these challenges, some clinicians undertook preoperative education in their preadmission clinic to help patients understand and weigh up the risks and benefits of pain medications. They argued that educating nursing staff, junior and senior medical staff, and general practitioners (GPs) was also essential to ensure cohesive pain management. Some clinicians attended conferences to stay informed about new pain management approaches, while others focused on improving self-awareness and seeking feedback from colleagues regarding opioid use and alternatives.

### Patient perspective themes

Detailed information about the patient themes, subthemes and supportive quotes can be found in Supplementary Appendix 6. They are summarised below.

### Theme 1: (unfulfilled) expectations of careful pain management

#### Patient expectations prior to surgery

Patients who were due to undergo total knee or hip replacement anticipated that their surgery would cause substantial pain, and that it would be appropriate to use the most powerful pain medicines available, which they believe to be opioids. Patients correctly expected that they would receive an opioid pain medicine post operatively but there was a strong expectation that any opioid would be prescribed with great care, and that patients would be closely monitored due to the high-risk nature of these medicines, illustrated by this quote: “*They're very careful these days, I think, and very aware, and keep an eye on things”.* Many felt that their doctor would only prescribe what was necessary, and that the decision would consider their unique circumstances “*I think my surgeon and my anaesthetists will send me home with what they think I need”*.

#### Subsequent patient experiences after surgery

These expectations did not align with reports from patients reinterviewed after their surgery. These patients were surprised by how little information or monitoring they received. They reported that their discharge pain medicines were prescribed with a “tick box” or “production line” approach, and that there were very few opportunities to ask questions along the journey of care through to discharge.

### Theme 2: risk-benefit trade-offs: fear of pain vs. fear of side effects

Patients had a good but incomplete understanding of the risks of opioids. This knowledge was acquired from their previous experience of side effects, internet searches, their social circles, and media such as documentaries or newspaper articles. Patients had seldom acquired this information from healthcare professionals during their care pathway. Many noted that addiction was a risk for some, but did not believe they were at risk themselves “*Top one [risk] would be addiction, except I don't think that's a risk for me, you know, like I'm 72 nearly and I've kind of managed my life so far”*.

Patients who aimed not to take all of the dispensed opioids rarely mentioned what they would do with leftover opioids (e.g., disposing or returning to a pharmacy). No one addressed potential harms in retaining some for future personal use or diversion.

Patients were generally anxious about their upcoming surgery, reporting fears regarding intense unmanaged pain, and the threat of not being listened to or believed when communicating about their pain. They also reported fears about the side effects and risks of all pain medicines, especially opioids but including non-steroidal anti-inflammatories and paracetamol. Consequently, most patients felt that it would be ideal to avoid opioids completely, but had low expectations for non-opioid pain medicine effectiveness and believed that only opioids would be able to offer the level of post-surgical pain relief that they might require. Thus, some undesirable side effects might be unavoidable.

### Theme 3. variation in self-management of opioid use after surgery

In the post-surgical follow-up interviews, many patients felt that they had to self-manage their opioid medicines due to receiving vague instructions to take them ‘minimally’, ‘for a short time’ or ‘wean when you are able’ without specifics. All patients reported that they were following these instructions, but there was ample room for (mis)interpretation. This is demonstrated in the following two quotes: “I don't remember getting any info at discharge regarding my medicines. I was just given rest of the box, with no chance to ask questions” and “My doctor said at discharge to wean myself when feeling ready, but gave me no other information”.

This may have contributed to the variation in how these patients used prescribed opioids after discharge from hospital. Some patients explained that they did not take any opioids, in line with their pre-operative goal. Others took multiple opioid tablets per day for more than six weeks. Patients did not directly link their decisions about duration of opioid use with the amount of pain they experienced. Those who took the opioids for multiple weeks reported using them as a sleep aid, or taking them prophylactically due to fear of pain if they stopped. A few explained that if their doctor prescribed medication it was important that they take it. Some likened opioids to antibiotics where the entire prescription should be finished.

## Discussion

This study provides new insights into the perspectives of both patients and prescribers regarding post-surgical analgesia following THR and TKR. Our findings highlight key themes in prescriber and patient perspectives on post discharge pain management, including mismatches between prescriber and patient perspectives, incomplete understanding or use of available knowledge by both parties, and being challenged by delicate risk-benefit balances.

### Interpretation of findings

#### Mismatch between expectations and practice

Our findings show a mismatch between patient expectations and prescriber practices. Prescribers described considerable variability in opioid prescribing, influenced partially by clinical experience, but also by contextual norms (e.g., what is usually prescribed at that site). They also described inconsistent handover to community care [e.g., to a general practitioner (GP)]. This was in stark contrast to patients’ expectations that their prescriptions would be highly individualised (based on their unique circumstances and risk profile) with clear instructions, and they would be closely monitored. Patients reported they trusted their doctors to prescribe only what is necessary due to the high-risk nature of opioids. However, this clashed with some prescribers’ tendency to prescribe opioids at a higher dose or frequency than what the patient may require.

All 6 patients noted in their post-surgery interviews that their expectations of personalised prescriptions, clear instructions and close monitoring were not met. There was substantial confusion about how to safely use their medicines, which led to large variation in how patients self-managed their medicines. For example, one participant reported “*I didn't feel that 5 weeks was too long to take the Endone. I think that's pretty good”*, while another reported “*I would think that 5 or 6 days of opioids is plenty. Certainly no more than 1 week”.* We noted that no patient mentioned using their pain level to guide how much opioid to take and for how long, but instead seemed to be attempting to interpret the prescriber's instructions and follow them correctly.

#### Guideline adherence

Many prescribers were either unaware of, or chose not to follow, prescribing guidelines. This could be because guidelines offer prescribers little in the way of specifics on doses and amounts of opioids to prescribe, likely because there is scant primary evidence for major orthopaedic surgeries on which to base recommendations (17, 18).

#### Institutional norms

Junior doctors reported basing their prescriptions on the ‘norm’ at each site, whereas senior doctors relied more on their clinical experience. Junior doctors tend to be the lead prescribers in public hospitals, where site norms appeared to be mostly in line with current guidelines (e.g., 10 or 20 tablets of short-acting opioids). In contrast, senior doctors manage prescribing in private hospitals where prescriptions were generally reported to be of higher amounts and included long-acting opioids, which guidelines specifically recommend against (18). Other factors such as financial constraints, hospital formulary restrictions, and variations in clinician training also contributed to differing opioid prescribing patterns between public and private settings.

#### Risk perception and knowledge gaps

Like prescribers, patients were also imperfectly informed. While most reported a good knowledge of the risks, many felt that the risk of addiction and dependence was not relevant to them personally and this may contribute to unintentional opioid overconsumption. They also lacked understanding of the risks of keeping unused opioids in their homes which can result in inappropriate opioid use both in the patients and their families when patients develop pain for other reasons. Our findings show that both prescribers and patients are challenged by the many risk-benefit balances they must straddle. Prescribers are balancing pain management with risks that they felt were even more delicate due to the population undergoing THR and TKR being generally older. Patients are balancing their fears of being in pain and being caught without adequate pain medicines with the side effects of opioids.

### Comparison to the literature

Other research has also highlighted the struggles of achieving a risk-benefit balance. One study found that many patients prefer having extra opioids prescribed to them as a precautionary safety net (19). Another study found that despite 96% of patients reporting adequate pain control with a reduced opioid regimen, they still expressed a preference for having access to “more than enough” opioids, reflecting fears that they may not be able to manage their pain effectively at home (19). Many patients expect to receive an opioid pain medication after surgery and are aware of potential for addiction, but do not associate opioid-dependency with prescribed opioids after surgery (20). These areas of discordance between knowledge, preferences and expectations can create confusion and unwarranted variation in how patients self-manage their pain medicines.

Previous studies have explored the factors influencing opioid prescribing among surgeons in countries affected by the opioid crisis, such as the United States. Several studies have found that prescriber attitudes are influenced by factors such as the surgeon's age and experience, a perceived ability to identify patients who may misuse opioids, and a concern about under-treating pain, which could result in patient complaints or the need for additional out-of-hours care (21, 22). However, our findings indicate that concern for patient complaints was not a major barrier to opioid reduction among Australian prescribers, particularly in public hospitals where most of our interviewed clinicians worked.

### Strengths and limitations

This qualitative study highlights factors that contribute to differences in opioid prescribing, providing context for future policy development. Triangulation of data from patient and prescriber perspectives, including post-operative follow-up with patients, provides a rich snapshot of mismatched perceptions, unfulfilled expectations and variation in opioid prescribing and usage. We identify gaps in education and communication, and the need for improved preoperative counselling and discharge education to enhance opioid stewardship and align pain management practices with patient needs.

The lack of interviews with GPs is a limitation, particularly given that GPs look after patients once they are discharged from hospital care and have a key role to play in patients’ ongoing medical needs. Previous qualitative studies involving GPs have found that they would like more direct communication between the surgeon and the GP to know whether post operative opioids should be tapered or continued (23). Additionally, the study lacked participants from rural areas, where access to GPs and pharmacists, and pharmacies may be more limited. Patients in metropolitan settings reported confidence in their ability to access additional pain relief if needed, but this may not be representative of patients across Australia. There may also be selection bias, as patients who agreed to be re-interviewed may have had a more positive or negative experience than the general population.

### Future directions

Future efforts could focus on better understanding the large variability in the number of opioids consumed following discharge that was reported by our participants. It is unclear whether this variation is due to a lack of instruction, leading patients to interpret appropriate use differently, or whether it truly reflects differences in pain experiences and analgesic needs. Further research could also examine how geographic disparities impact post-surgical pain management and opioid accessibility.

The discrepancies between prescriber intentions and patient experiences underscore the importance of structured, patient-centred communication regarding pain management. Preoperative counselling and improved discharge education may help align patient expectations with safe and effective opioid use. It is unclear whether basic education is not occurring, or if patients cannot recall it, perhaps due to the timing, brevity, or verbal nature (not written) of the delivery. For example, patients could be educated that the number of tablets prescribed on discharge is more than what is required for most patients and further prescriptions can be dispensed at the follow-up appointment if needed. Information on safe disposal of leftover opioids would also be required. Patients could also be educated that they should avoid opioid analgesia unless the pain is causing impairment of activities of daily living, as opposed to taking it to eliminate pain as part of instructing patients to take opioids minimally. Furthermore, a greater emphasis on post-discharge follow-up, particularly in private healthcare settings where access to specialist pain management may be limited, could enhance patient outcomes, and reduce reliance on opioids.

The unwarranted variability may be improved by guidelines providing more specific recommendations. This may assist prescribers in providing clear, evidence-based recommendations to their patients. To inform such guidelines, robust clinical trials need to be done to test various amounts and doses of pain medicines prescribed to people, using various means of individualising the prescription, to determine what practices offer the best benefit-harm balance. Currently, there are no trials comparing opioids to non-opioids after major surgeries (24), and a only a few comparing higher vs. lower doses (25).

Furthermore, future research should consider non-opioid options for pain management after major surgery including new compounds like Suzetrigine which is a non-opioid selective inhibitor of sodium dependent pain signalling pathways in peripheral nerves and which has been recently approved in the US for the management of acute pain (26).

## Conclusion

Our study highlights tensions between prescriber and patient expectations, concerns, and practices for managing pain after discharge from TKR and THR. Patients expect individualised, highly cautious prescribing but this is not often what occurs in practice. Prescribers and patients both have incomplete knowledge and lack of reliable information. Both parties are challenged by the delicate risk-benefit balances that underpin their decisions. Addressing these challenges requires a multifaceted approach, including deepening the evidence-base to inform guidelines, providing prescriber and patient education, and improved communication. By integrating patient and prescriber perspectives, we can move toward a more balanced, safe, and effective approach to post-surgical pain management.

## Data Availability

The datasets presented in this article are not readily available because no data other than what is included in this article or Supplementary Material will be shared. Requests to access the datasets should be directed to Ian Liang, ian.liang@health.nsw.gov.au.
