# Supplementary material for: Expectations about pain management after discharge from total hip and knee replacement surgery: a qualitative study with patients and prescribers
Source: Front Pain Res (Lausanne). 2025 Sep 24;6:1647020. doi: 10.3389/fpain.2025.1647020 (PMC12504195; doi:10.3389/fpain.2025.1647020)
Supplement: Supplementary file 1 [file Table1.docx]

Demographic Questions - Prescribers

1. Are you involved with prescribing (including supervising others prescribing) pain medicines to people upon discharge home after total hip or knee replacement? (Yes, No)
2. What is your age?
3. How long have you practiced medicine (not including medical school)?
4. What postcode do you mainly work in?
5. What is your gender?
6. Which health system do you work within (Public, Private, Both)
7. What is your professional role (Orthopaedic surgeon, Orthopaedic registrar, Orthopaedic Junior Medical Officer, Anaesthetist, Anaesthetic Registrar, Geriatrician, Rehab physician, Other)
