# Supplementary material for: Expectations about pain management after discharge from total hip and knee replacement surgery: a qualitative study with patients and prescribers
Source: Front Pain Res (Lausanne). 2025 Sep 24;6:1647020. doi: 10.3389/fpain.2025.1647020 (PMC12504195; doi:10.3389/fpain.2025.1647020)
Supplement: Supplementary file 2 [file Table2.docx]

Demographic Questions – Patients

1. Are you scheduled for, or on the waiting list for a total hip replacement?
2. Are you scheduled for, or on the waiting list for a total knee replacement?
3. When is your surgery scheduled for?
4. How long have you had pain in your hip or knee?
5. What is the reason that you are having a total hip or knee replacement?
6. Have you previously used opioid pain medicines for your hip/knee pain?
7. What is the postcode of where you live?
8. What is your age?
9. What is your gender?
10. What country were you born in?
11. What is the main language that you speak at home?
12. What is your proficiency in spoken English?
13. Are you an indigenous Australian?
14. How would you rate your average pain over the previous week (0 is no pain, 10 is the worst imaginable pain)
15. Which health system will you access for your hip or knee replacement?
