# Supplementary material for: Expectations about pain management after discharge from total hip and knee replacement surgery: a qualitative study with patients and prescribers
Source: Front Pain Res (Lausanne). 2025 Sep 24;6:1647020. doi: 10.3389/fpain.2025.1647020 (PMC12504195; doi:10.3389/fpain.2025.1647020)
Supplement: Supplementary file 3 [file Table3.docx]

Interview questions – Prescribers

Opening spiel:

Thank you for volunteering your time to help with this research. We are interested in understanding what is guiding the complex decision about what to prescribe to a person for pain upon discharge after a total knee replacement. I’ll be asking you some questions which are designed to try and get at the process of the decision and the factors that doctors might be weighing up as they make them. If you are comfortable, I will record our conversation so that I can transcribe the interview, and then delete the recording. I am only interested in the audio however your video will automatically be recorded if you leave it on. It is completely fine whether you prefer to keep the video on or turn it off. Do you have any questions about the study before we begin?

Opening question/ice breaker:

1. How long have you been doing this kind of work?

TDF based questions:

1. What kind of information do you draw upon to help you make decisions about analgesic prescribing upon discharge after TKR?

*Prompts:*

- *Are you aware of any guidelines or resources that doctors use to help them with these decisions?*
- *Are there any ‘rules of thumb’ you call upon?*

1. What sorts of skills are needed to make sound clinical judgements about prescribing analgesia upon discharge after TKR? Where do these skills come from?
2. As you are aware, it can be hard to relieve pain (and anxiety about pain) while also complying with opioid guidelines. How do you manage the tensions in those professional obligations?

*Prompts:*

- *Do you believe it’s possible to prescribe a regimen that both manages pain effectively and complies with guidelines/minimises risk of harm?*
- *What are the major challenges? How do you navigate them?*

1. Some US studies found that doctors sometimes prescribed opioids more than they would like because of concerns that undertreating a patients’ pain could result in them requiring care out of hours, representing to hospital or lodging a complaint. In your experience, how pressing are those concerns in the Australian context?
2. Doctors often talk about the balancing act of prescribe pain medicines regimens that manage pain adequately, without eliciting side effects like sleepiness or nausea that might impair the person’s ability to complete activities like rehab. What particular factors are you trading off when prescribing for post-surgical pain? Does it differ with opioids?
3. How do you view the state of pharmacology when it comes to pain management? Are there good options available to you? Under what circumstances are opioids are required in the regimen?
4. There are lots of different views out there about prescribing for pain. To what extent is your practice shaped by the opinions of patients and your colleagues?
5. In what ways do your past experiences with patients in managing their pain influence your current practices? We’re wondering if there are any cases in particular that really shaped your practice.

Closing question

1. We are thinking about conducting a randomised controlled trial comparing a ‘standard’ versus a ‘low’ dose of opioids (with both groups also receiving a non-opioid regimen of paracetamol and NSAIDS) prescribed at discharge. We hypothesis that a lower dose of opioids might be just as effective on pain, and perhaps have less side effects, than the standard dose. What do you think about this idea?

Prompts:

- *What is the ‘standard’ discharge regimen in your work setting?*
- *Do you see the value in comparing two doses of opioids? Or would you rather see an opioid versus non opioid regimen compared?*
- *What factors or challenges do you foresee with referring patients into such a trial?*
- *Is there any information or resource that you think might help with recruitment?*

Closing comments:

Thank you very much for your time. Do you have any closing comments or questions?
