# Supplementary material for: Expectations about pain management after discharge from total hip and knee replacement surgery: a qualitative study with patients and prescribers
Source: Front Pain Res (Lausanne). 2025 Sep 24;6:1647020. doi: 10.3389/fpain.2025.1647020 (PMC12504195; doi:10.3389/fpain.2025.1647020)
Supplement: Supplementary file 4 [file Table4.docx]

Interview questions – Patients

Opening spiel:

Thank you for volunteering your time to help with this research. We are interested in understanding what people are considering when deciding what to ask for, or accept for, pain medicine when they are discharged from hospital after a total hip or knee replacement. I’ll be asking you some questions which are designed to try and get at the process of the decisions and the factors people might be weighing up as they make them. If you are comfortable, I will record our conversation so that I can transcribe the interview, and then delete the recording. I am only interested in the audio however your video will automatically be recorded if you leave it on. It is completely fine whether you prefer to keep the video on or turn it off. Do you have any questions before we begin?

Opening question/ice breaker:

1. Could you tell me a bit about your condition and how you have been managing your pain so far?
2. Will this be your first time having surgery?

TDF based questions:

1. So you’re scheduled for a total knee replacement in the next X weeks, what do you expect will happen with pain management after you leave hospital?
2. What kind of information (if any) do you have about how pain is usually managed, or should be managed, once you’re home after your surgery?

- *Have you already discussed with a doctor / other health professional?*

1. Do you feel able to discuss your needs/expectations/concerns about pain medicines after you leave hospital with the doctor?
2. How confident are you that you will be able to manage your pain satisfactorily after leaving hospital?
3. What is your understanding of the risks of taking pain medicines? Are there any risks in particular that you think might influence your choice about what/how much to take or ask for?

- *Do you have any particular concerns about opioid pain medicines? (Opioid medicines are a class of drug which includes Endone, morphine, etc. They are thought of as being strong pain killers but they have substantial side effects such as constipation, nausea, sleepiness, and serious side effects too like addiction and risk of overdose).*
- *Do you feel you have access to the information you need to make an informed choice?*

1. Sometimes people have to balance pros and cons in making decisions about medicines. Are there any ‘trade-offs’ that you are considering?

- *E.g. how well the pain medicine works compared to its side effects?*

1. There are lots of different views out there about using medicines for pain. To what extent is your opinion shaped by the views and experiences of your friends and family?
2. In what ways do your past experiences managing pain influence your current beliefs or plans? We’re wondering if there are any experiences in particular that really shaped your view on the topic.

Closing question

1. We are thinking about conducting a research study that I’d love to get your opinions on. We want to compare two different kinds of pain management for people who are discharged from hospital following a total knee replacement. One group would get a ‘standard’ dose of opioids, the other would get a ‘lower than usual’ dose of opioids (with both groups also receiving other non-opioid pain medicines). We expect that a lower dose of opioids might be just as effective on pain, and perhaps have less side effects than the standard dose. What do you think about this idea?

Prompts:

- *What concerns would you have about joining a study where you might be put in the group which gets a ‘lower’ dose of opioids (on top of other medicines)? Would your concerns stop you from joining the study? What sort of information could reassure you that it’s OK to be in this group?*
- *We’re also thinking of having a group that receives no opioids at all (just other types of pain medicines). What concerns would you have about joining a study where you might be put into that group? Would your concerns stop you from joining the study? What sort of information could reassure you that it’s OK to be in this group?*

Closing comments:

Thank you very much for your time. Do you have any closing comments or questions?

Follow up interviews:

1. What information did you receive since we last spoke? Did you attend pre op appointment?
2. Did it make you feel any differently?
3. (if has surgery) did your concerns and expectations line up well with what you actually experienced after you discharged home?
4. How many and which pain medicines did you end up using?
