# Supplementary material for: Expectations about pain management after discharge from total hip and knee replacement surgery: a qualitative study with patients and prescribers
Source: Front Pain Res (Lausanne). 2025 Sep 24;6:1647020. doi: 10.3389/fpain.2025.1647020 (PMC12504195; doi:10.3389/fpain.2025.1647020)
Supplement: Supplementary file 5 [file Table5.docx]

**Appendix 5: Results table from prescriber interviews, themes, subthemes and quotes.**

| Subtheme | Quote |
| --- | --- |
| **A patchwork of prescribing practices** |  |
| **What works and why?** |  |
| Clinicians saw the role of post-surgical pain relief to support rehabilitation, improve function and enable sleep, rather than just eliminate pain. | Anaesthetist: *Using pain scores has been problematic. Analgesia should enhance activity or movement, not just relieve pain at rest.* |
| Some surgeons followed strict protocols, while others deferred to anaesthetists or pain teams. | Geriatrician: *There’s a lot of variety in analgesic prescribing because the patient is under the orthopaedic team, and they have different preferences. Where I’m working, for a particular surgeon for their total knee replacements there’s a strict protocol that they want, pregabalin, Panadol and others.* |
| Most patients received simple analgesia with regular paracetamol and a long-acting NSAID like celecoxib or meloxicam. | Orthopaedic surgeon: *With paracetamol and anti-inflammatories, their effectiveness is just as good, but their risk is lower than opioids.*  Anaesthetics registrar: *Patients should have regular paracetamol and nonsteroids for up to seven days following surgery, especially COX2 inhibitors which have less gastric side effects.* |
| Medications like pregabalin or gabapentin were preferred as an adjunct by some clinicians for neuropathic pain because of perceived fewer side effects. | Rehab physician: *We generally prescribe paracetamol along with medications like pregabalin or gabapentin for neuropathic pain, tapering those off more slowly.* |
| Some clinicians recommended adjunct complementary therapies like fish oil and turmeric, despite admitting that their effectiveness remained unproven | Geriatrician: *Some alternative medicines have also been shown to have an anti-inflammatory effect like turmeric and fish oil. I think it does have some evidence and at the very least they’re getting some level of placebo effect and there’s quite a very little chance of an adversary.* |
| Ice packs were commonly used in hospital and at home. | Geriatrician: *We use a lot of ice particularly inpatient, but when they go home as well especially for the knees although it doesn’t work quite so well in hips.* |
| While most patients received additional opioids prescribed on an ‘as required’ basis, prescribing practices varied in terms of the type of opioid, dose, frequency, and duration. | Orthopaedic JMO: *In the post-operative orthopaedic setting, most patients will require some level of opioid pain management alongside non-opioid options.*  *Rehab physician: If patients are discharged without adequate pain management, they may struggle to participate in rehab.* |
| **How much is enough?** |  |
| Some junior medical officers prescribed 10-15 tablets, while others prescribed up to 20 tablets, particularly for early discharges. | Orthopaedic JMO: *Typically, we prescribe 10 tablets of Endone or Tapentadol and they will follow-up with their GP in two weeks.*  Anaesthetist: *Pharmacists stock oxycodone in boxes of 20, so I typically write for that amount. It's practical and convenient.* |
| The instructed frequency of use varied between up to q3 to q12 hourly, although the clinician would often advise patients to use less than the maximum if they were able to manage their pain. | Orthopaedics JMO: *During medical reconciliation, we typically copy the doses prescribed by the anaesthetist. Despite prescribing at q3 hourly, by the time the patient goes home, they typically won’t use the medication strictly every three hours. My general approach is that the 10 tablets should last them a few days until they can see their GP for a new prescription for opioid analgesia.* |
| Opioid use duration was seen to depend on recovery. | Rehab physician: *Most patients receive pain management during rehabilitation and are discharged with PRN-only prescription.* |
| Anaesthetists typically limited opioids to one week’s supply before transitioning to non-opioid relief. Others extended use for up to six weeks, particularly after knee replacements which they considered to be more painful. | Anaesthetic Registrar: *By day five or six post-op, they generally transition to non-opioid pain relief.*  Orthopaedic surgeon: *For knee replacements, I might prescribe opioids by week 6 about one in ten times. It would be uncommon for a hip patient to need any opioid medication.* |
| Clinicians explained that challenges arose when patients had unrealistic expectations of how effectively joint replacement surgery would relieve their pain, resulting in prolonged use of opioids in some cases. | Orthopaedic surgeon: *Some have a high expectation of a pain-free joint, so they struggle when the pain is still there after surgery.* |
| **Private vs Public Variation** |  |
| Clinicians in private hospitals often prescribed higher opioid doses due to perceptions of greater patient expectation of pain relief. In contrast, public hospital clinicians were treating patients with more advanced disease who had been waiting for surgery for longer periods of time. Clinicians argued that these patients were more accustomed to managing pain and could thus manage post operatively with fewer opioids due to a higher pain threshold. | Orthopaedic surgeon: *I would probably say that private patients tend to use opioids more. Public patients generally have worse disease and have been dealing with pain for a lot longer, so they might accept their pain more readily than private patients. Private knees or hips tend to be less advanced than public cases.* |
| In private hospitals, anaesthetists typically handled discharge prescriptions, whereas in public hospitals, junior medical officers (JMOs) were responsible for most prescriptions. | Anaesthetist: *In the public sector, a big challenge is that I'm not always the one writing the discharge prescription. Often, it’s the intern on the surgical ward who does it, and they may not have as much experience.* |
| In both hospital settings, there was a lack of consistency regarding analgesic prescribing with individual variations as well as variations between disciplines. | Orthopaedic JMO: *There's a discrepancy between certain surgical registrars and the acute pain service regarding the use of long-acting opioids. Some registrars advocate for their use when patients report significant pain, whereas the acute pain service tends to be hesitant about prescribing them in acute post-operative settings.*  Geriatrician: *The challenge is having so many clinicians involved, each with their own approach to pain management.* |
| The higher cost of tapentadol resulted in it being excluded from the formulary in one public hospital, leading to more frequent use of less expensive alternatives like oxycodone. | Anaesthetist: *Endone is cheaper and easier to use, whereas Tapentadol is often restricted (in public hospital) due to cost.* |
| Public patients usually received specialist pain clinic follow-up care, whereas private patients tended to rely on general practitioners (GPs) for analgesia post-operatively. | Anaesthetist: *[In the public sector] we follow up with patients through our pain clinic after discharge. In the private sector, often patients are discharged without the same level of follow-up unless they’re referred to a GP or a pain specialist. They usually only follow-up with their orthopaedic surgeon.* |
| One orthopaedic surgeon noted that GPs often lacked adequate training in post-surgical management and may feel disempowered to reduce opioids, especially when they are not involved from the start of the patient's treatment. | Orthopaedic surgeon*: I feel opioids are overprescribed in the community in that a lot of GPs continue whatever’s prescribed in hospital.*  Orthopaedic surgeon: *I don’t think GPs are very experienced with [opioid prescriptions following a total hip or knee replacement].* |
| **What Counts as Evidence for Practice?** |  |
| **Variable use of guidelines** |  |
| Clinical guidelines on post-surgical pain management, particularly regarding opioids, were often criticised by senior clinicians for being based on low-quality evidence. | Rehab physician*: Guidelines are aspirational, but at the bedside, clinicians make decisions based on experience.*  Anaesthetist: *The issue with hip and knee replacements is that there’s a lot of literature out there, but a lot of it is low-quality evidence, so it can be hard to find definitive guidance.* |
| Some anaesthetists, recognising this limitation, often made decisions based on personal experience rather than guidelines. | Anaesthetist: *There are guidelines, but guidelines can only go so far* |
| Despite being responsible for most opioid prescriptions on discharge in public hospitals, JMOs in our study were less familiar with the latest guidelines. | Orthopaedic JMO: *I'm responsible for prescribing about 95% of the tie, but I escalate if the patient is in significant pain.*  Orthopaedic JMO: *We follow what the anaesthetist prescribes, but we don't always have access to the latest guidelines.* |
| Some core messages from guidelines still influenced prescribing, especially the preference for atypical opioids such as tapentadol and for using the lowest effective opioid dose. | Anaesthetist: *Historically, I used to prescribe a combination of panadeine forte with oxycodone. However, these days, I’m moving away from oxycodone and instead using Tapentadol or other alternatives where possible.* |
| **Practice-Based Knowledge** |  |
| Many clinicians conveyed that they relied on professional experience or guidance from colleagues when making prescribing decisions. | Orthopaedic JMO: *It’s a mix of experience and guidance from others. Sometimes it’s trial and error with patients – figuring out what works and what doesn’t. I also rely on advice from my seniors or the APS.* |
| Preferences for or against certain types of analgesia such as NSAIDs or opioids often stemmed from previous encounters with side effects or complications in patients. | Geriatrician: *I’ve seen a lot of adverse events, sedation, needing ICU, needing prolonged intubation because of opioids. That scares me. That’s why I’ve shaped my practice into very minimal analgesia because I’ve seen, very, very bad outcomes.* |
| Some senior clinicians preferred slow-release opioids for long-lasting pain relief despite acknowledging that guidelines advised against their use. | Rehab physician: *The Faculty of Pain Medicine released guidelines against slow-release medications for opioid-naïve patients post operatively. I believe patients are better managed on slow-release medications while in the hospital, as it helps them engage in therapy. We use quick-acting medication for breakthrough pain, rather than having them take immediate-release medication multiple times a day.* |
| JMOs, who often have limited experience managing pain after joint replacements, rely on their registrars or prior experience prescribing analgesics during non-orthopaedic rotations. | Orthopaedic JMO: *Being an intern, a lot of your learning comes from informal advice from seniors.* |
| Senior anaesthetists had found advances with regional techniques and medications like tranexamic acid to have reduced overall opioid use. | Anaesthetist: *Analgesic requirement for hip and knee replacements have dramatically reduced. This is due to factors like the use of tranexamic acid.* |
| **Risk/Benefit Trade-Offs: pain, side effects and patient expectations** |  |
| Geriatricians were especially cautious with opioids due to concerns about sedation and delirium, which can worsen patient outcomes. They noted however, that untreated pain itself can cause delirium. | Geriatrician: *For elderly patients in their 90s, I would not give a large dose of opioids due to concerns about respiratory depression.*  Geriatrician: *Delirium in an acute setting can lead to disability and higher chances of long-term care needs.* |
| The risk-benefit assessment depended on patient-specific factors such as chronic pain, comorbidities and overall health. For example, NSAIDs like meloxicam were used safely in patients with normal renal function, but alternatives like hydromorphone may be preferred in those with renal disease. | Anaesthetist: *If the patient has CKD, hydromorphone is preferable, and we avoid NSAIDs.* |
| A patient’s age, weight and co-morbidities such as sleep apnoea—which raises the risk of respiratory depression—were also important considerations. | Anaesthetist: *Patients who have sleep apnoea or who are obese, they need to have more cautious prescribing because they’re more sensitive to opioids. Patients who have renal failure and the elderly are also a special category that needs more caution with prescribing opioids on discharge.* |
| Clinicians did not tend to reduce opioids if side effects could be managed with other measures such as aperients for constipation. | Geriatrician: *When it comes to things like constipation or other side effects, I guess, managing those with alternatives, aperients, and things like that. So, I wouldn't trade off pain relief for those less sinister type of side effects.* |
| Surgical factors, such as recovery from total knee replacements being viewed as more painful than total hip replacement, lead to increased dosage and duration of opioid prescriptions. | Orthopaedic surgeon: *Knee replacements tend to be more painful [compared to hip replacements], and 15-20% patients report dissatisfaction two years down the line.* |
| Clinicians observed that patient expectations significantly influenced their risk-benefit assessments. Some patients, particularly those with high expectations of minimal or no pain, may request more opioids, leading to overuse. | Orthopaedic surgeon: *Younger males often have a higher pain threshold pre-surgery but struggle more post-surgery. They end up dissatisfied as they didn't anticipate how tough recovery would be.* |
| Anxious patients or those with prior negative experiences with pain, often demanded more opioids and reported higher pain levels, possibly due to a lower pain threshold. | Anaesthetist: *Those patients who are more anxious, they tend to feel that they require more opioid analgesic agents. They’re often the ones that get into trouble with adverse effects.* |
| One geriatrician noted that orthopaedic surgeons played a significant role in managing expectations as patients are more likely to follow pain management plans when surgeons emphasise the need to reduce opioid use when appropriate. | Geriatrician: *With one hip patient, the surgeon’s word has been gold for her. The surgeon said no opiates and she’s just gone ahead and managed with no opiates.* |
| **Addressing challenges** |  |
| To address these challenges, some clinicians undertook preoperative education in their preadmission clinic to help patients understand and weigh up the risks and benefits of pain medications. | Anaesthetist: *We educate patients before surgery, letting them know pain will not be zero and that managing pain for functionality is the goal.* |
| They argued that educating nursing staff, junior and senior medical staff, and general practitioners (GPs) was also essential to ensure cohesive pain management. | Anaesthetist: *There’s a need for more education, both for patients and healthcare professionals. Pain management should be a collaborative effort, involving the GP, pain management specialist, and the surgical team. It shouldn’t be done just by one professional in isolation.* |
| Some clinicians attended conferences to stay informed about new pain management approaches, while others focused on improving self-awareness and seeking feedback from colleagues regarding opioid use and alternatives. | Geriatrician: *From an individual level, upskilling, going to conferences, talking to others, self-awareness, and the want to improve your practice can overcome some barriers.* |
