# Supplementary material for: Expectations about pain management after discharge from total hip and knee replacement surgery: a qualitative study with patients and prescribers
Source: Front Pain Res (Lausanne). 2025 Sep 24;6:1647020. doi: 10.3389/fpain.2025.1647020 (PMC12504195; doi:10.3389/fpain.2025.1647020)
Supplement: Supplementary file 6 [file Table6.docx]

**Appendix 6: Results table from patient interviews, themes, subthemes and quotes.**

| Subtheme | Quote |
| --- | --- |
| INTERVIEWS n = 13 pre surgery and n = 6 followed up post surgery | |
| **Patients expected personalised prescriptions, clear instructions and close monitoring, but this did not eventuate** | |
| Most are expecting to be given something strong for short term use | *“I would expect to have Endone.”* |
| Many were expecting clear instructions, and close monitoring given the known risks, but this clashes with other’s experience when they felt they didn’t receive information after past surgeries | *“So, but I would also think that the pain means that they dispense would not be that kind of medication that you could become dependent on all. There'd be very strict instructions, anyway, about you know how much when you take it and how much you take, and for how long”*  *“They're very careful these days, I think, and very aware, and keep an eye on things.”* |
| Many expected that their prescriber will consider their individual needs resulting in the misbelief that if pain medecines were prescribed, they were being recommended because of their unique circumstance. | *“I think my surgeon and my anaesthetists will send me home with what they think I need”* |
| Actual experiences (reported in post-op interviews) were that most were given minimal information or discussion, in “mechanical” / “tick box” / “production line” manner, and expected to self-manage | *“I don’t remember getting any info at discharge regarding my medicines. I was just given rest of the box, with no chance to ask questions”*  *“My doctor said at discharge to wean myself when feeling ready, but gave me no other information”* |
| One person interpreted this as 6 weeks of taking opioids daily as being “good”, others interpreted as ideally getting off them as soon as possible or not even starting | “I didn’t feel that 5 weeks was too long to take the Endone. I think that’s pretty good”  *compared with*  *“I would think that 5 or 6 days of opioids is plenty. Certainly no more than 1 week”* |
| **Strong but incomplete awareness of opioid risks and different interpretations of vague instructions** | |
| Good general knowledge about the risks | *“I expect some sort of tummy problems. I would expect, maybe a bit of constipation. and I will be drinking a lot of water, because I'll be worrying about my kidneys. yeah, I do worry about my internal organs”* |
| Previous experience had informed their knowledge | *“I also was conscious of not getting addicted to them.”*  *“I know that the opioids is going to make me sleepy. but after a big operation I don't think it helps.”* |
| Only one person mentioned correct disposal of leftover opioids | “*Yeah, I think they would just take it till they finish till they finish the packet, but I don't like the feeling, you know, because it does knock you around. Which is why I felt like I had to sleep in the day. At the end of the day I hope that I will be a to go to the chemist and say, Here's some Endone, and I don't need it anymore.”* |
| Some did not use any opioids, some used 3 times a day and required multiple extra scripts of 20 tablets. Could this be because of lack of instruction and therefore people interpreting “appropriate/safe” use differently, or is it true reflection of different pain experiences and therefore different needs? | *“I was given Endone PRN 20 tablets, which I used 3 per day, then 2, then 1. I rang the hospital and was given another script for 20 Endone. Then saw my GP at 4 weeks and was given Panadeine Forte but became nauseous, so was given another 20 Endone even though I still had 10 left.”*  *“I was discharged with only Brufen & asprin”* |
| Multiple people had undergone major musculoskeletal surgeries and chosen not to take opioids after discharge as their pain was not troublesome enough. Generally, people who didn’t need them in the past were expecting not to need them (or need very few) for their upcoming surgery. | *No direct quote available* |
| **Risk-benefit trade-offs: fear of pain versus fear of side effects** | |
| Fears about not being listened to or their pain being ignored as this had happened in the past. Therefore, many had concerns about lack of access to strong pain killers. | *“I'm not a sook. you know. Normally, I'd say, Yeah, whatever let's sort it out. And but I actually said, and I cried, I said, I'm so sorry for myself.”* |
| Most interviewees were fearful about the side effects and risks of opioids. Some had friends who had died or suffered from overdose or addiction. | *“the first week I was reacting to the morphine which no one knew. They just kept on giving it to me. I had massive headaches. I couldn't stand the bright light. I had to have the room darkened all the time. I slept. I had nausea”*  *“You've been given all these things which obviously helps, you know, initially. I'm not saying they don't help, but I am worried about it, because look when I came off that opioid thing I had withdrawal symptoms.”*  *“The Endone made me feel vague, confused, and strange.”* |
| Most describe not wanting side effects and risks of opioids, but think that is something they must suffer through for better function and sleep | *“I think maybe they do need more information about the side effects. But when you're in a lot of pain you just take them. You just don't care about anything but relief.”* |
| Wanting quick access to strong pain relief just in case, but hoping to not require it | *“I think I would probably go for something heavier just to take home because I don't know how it'll feel.”* |
| Most felt comfortable that they could access their GP and/or a pharmacist once discharged if they needed more pain relief. Howevef, we did not interview people outside of metropolitan areas. | *“It’s not easy to get in to see a GP quickly, but I could call in advance if needed.”* |
| Some believe that addiction isn’t a risk for them personally, although some knew others who had experienced addiction. | *“Top one would be addiction, except I don't think that's a risk for me, you know, like I'm 72 nearly and I've kind of managed my life so far”* |
